# Redo-redo aortic root replacement with a mechanical valved conduit in a patient with von Willebrand's disease: Case report

**DOI:** 10.1186/1749-8090-5-59

**Published:** 2010-08-13

**Authors:** Kasra Shaikhrezai, Usman Bashir, Sheena Millar, Julia Anderson, Edward T Brackenbury

**Affiliations:** 1 Department of Cardiothoracic Surgery, Royal Infirmary of Edinburgh, Edinburgh, UK; 2 Department of Haematology, Royal Infirmary of Edinburgh, Edinburgh, UK; 3 Department of Anaesthetics, Royal Infirmary of Edinburgh, Edinburgh, UK

## Abstract

A 40 year-old female, with a history of cardiac surgery for congenital aortic valve stenosis and von Willebrand's disease (VWD) presented with increasing shortness of breath due to mixed aortic valve dysfunction. With a paucity of such cases in the literature, we describe the successful outcome of a patient with VWD who underwent elective redo-redo aortic root replacement with a mechanical valved conduit. She was given a three-month trial of warfarin pre-operatively to evaluate the extent of bleeding risk. Her post-operative course was uneventful and she was discharged home after six days.

## Background

VWD is an autosomal dominant bleeding diathesis with an incidence of 2-3% in the general population. The disease is characterised by a partial quantitative decrease of qualitatively normal von Willebrand factor (VWF) and Factor VIII (FVIII) [1]. Recently researchers have reported that the increased shear stress resultant from a stenotic valve causes mechanical disruption and cleavage of VWF by ADAMTS-13, a metalloprotease enzyme that cleaves VWF, during passage through a stenotic orifice affecting the molecular conformation of large VWF multimers [2,3]. There is a small and evolving literature regarding the management of patients with VWD undergoing cardiac surgery. Our patient received a trial of warfarin preoperatively which is a challenging decision in the context of VWD disease. We performed Redo-redo aortic valve replacement with a mechanical valved conduit. The surgical procedure accompanied by haematologist and anaesthetist input is discussed as well.

## Case presentation

A 40 year-old female with type-I VWD and factor XII deficiency - a combination of haemostatic defects known as 'San Diego variant'- presented with exertional shortness of breath, tiredness and dizzy spells. Her basal FVIII/VWF: ristocetin cofactor and VWF Ag levels were 0.46 IU/ml and 0.40 IU/ml respectively.

At the age of 25 years, she had undergone a homograft aortic valve replacement (AVR) for congenital bicuspid aortic valve disease and severe aortic stenosis. Soon after surgery the implanted homograft became infected with *Streptococcus Viridans *causing vegetations and a paravalvular leak resulted in a re-do homograft AVR four months later. After the second operation she developed complete heart block and a permanent pacemaker was implanted. During these first two cardiac operations she received Haemate P concentrate (CSL Behring,UK Ltd) which is a plasma-derived FVIII concentrate rich in VWF, with a ratio of FVIII:C to VWF ristocetin cofactor of 1:2.2 and no major bleeding occurred. Since that time the patient had not suffered from major bleeding.

Regular follow-up in 2008 revealed that the implanted aortic homograft was degenerating. A trans-thoracic echocardiogram demonstrated mixed aortic valve disease with severe transvalvular regurgitation and a peak gradient of 59 mmHg accompanied by LV dilatation at 6.4 cm. LV systolic function was preserved with no hypertrophy and a mobile linear structure in the outflow tract suggesting prolapse of the cusp. A contrast computed tomography (CT) of the chest confirmed dilatation of the ascending aorta to 5 cm and it was appropriate to consider ascending aorta root replacement with a mechanical valved conduit. To evaluate whether the administration of vitamin K antagonists might pose bleeding problems post-operatively, she was warfarinised pre-operatively for a period of three months with an International Normalised Ratio (INR) range 2.0-3.0. After the trial period of warfarin had demonstrated no significant bleeding episodes, cardiac surgery was planned.

Following administration of 1000 IU FVIII/VWF concentrate prior to induction she was taken to the operating room where, under general anaesthesia, re-opening of sternotomy was performed. She was placed on cardiopulmonary bypass (CPB) via femoro-atrial cannula with full heparinisation at 300 IU/Kg and cooled to 32°C. The heart was densely adherent and required careful dissection. Aortic cross-clamp was applied distally beyond the dilated ascending aorta root and 500 ml of cold blood cardioplegia administered retrogradely into the coronary sinus to stun the heart. An aortotomy was performed and 1000 ml cardioplegia was delivered into the left and right coronary ostia. Most of the ascending aorta was dissected and removed including the sub-coronary inclusion homograft, which was heavily calcified. A size 25 mm Carbo-Seal Composite (Sultzer Carbomedics Inc, Austin TX) was implanted and coronary arteries on Carell patches re-attached to the neo-aorta. Cross clamp and bypass time were recorded at 132 and 156 minutes respectively. Post-bypass another 1000 IU of FVIII/VWF concentrate was given to ensure adequate replacement of VWF. Heparin was reversed in the usual manner with protamine 300 mg. The patient came off bypass uneventfully; however the suture lines continued to bleed due to a coagulopathic state confirmed by thromboelastography (TEG) requiring Fresh Frozen Plasma (FFP) and Bio-Glue (Cryolife Inc, Kennesaw GA). She was transferred to the Intensive Treatment Unit haemodynamically stable. Coagulation parameters are given in table 1.

**Table 1:** Pre- and post-operative hematological parameters

Coagulation profile	Pre-op	Post CPB	Discharge
**Hb** 115-165 g/L	117	77	106

**Activated partial thromboplastin time (APTT)** 26-36 secs	39	25	37

**INR**	1.2	1.2	2.6

**FVIII: C** 0.5 - 1.5 IU/ml	0.59	0.67	1.43

**Factor IX: C** 0.7-1.4 IU/ml	0.71	-	-

**Factor XII assay** 25 - 250 U/dl	16.4	-	-

**FVIII/VWF: ristocetin cofactor assay** 0.42 - 1.22 IU/ml	0.46	0.32	1.84

Pre-operative ristocetin induced platelet aggregation was normal with 79% aggregation at a ristocetin concentration of 1.5 mg/ml; VWF collagen binding was 43%

Two chest drains were removed safely on day one when the total blood loss was 2250 ml. In total, four units of packed red blood cells; three units of FFP and two units of platelets were given, in view of coagulopathy and anaemia. VWF levels were maintained above 100% throughout the operation and remained above 100% for over 5 days post-operatively without the need for exogenous factor administration beyond those already stated peri-operatively. Thromboprophylaxis using unfractionated heparin (25000 IU/2 ml) 5000 IU three times a day subcutaneously was commenced on post-operative day (POD) one and then she was warfarinised the same day aiming for an INR range of 2.0-3.0. Her post-operative course was uneventful and she was discharged home on POD 6 when her INR was within the therapeutic range. The patient was very well and asymptomatic six weeks later at a follow up visit with no bleeding or thrombotic events reported.

## Conclusion

Due to a previous satisfactory response to FVIII/VWF concentrate and contraindication of desmopressin in patients with cardiac insufficiency because of fluid retention [4], FVIII/VWF concentrate was chosen as the treatment of choice to prevent peri- and post-operative bleeding. The replacement therapy can be monitored by factor assays performed in a specialist haemostasis laboratory [5]. Such assays require a turnaround time of approximately one hour and are essential to enable optimal control of factor levels and dosing.

Clinically bleeding severity correlates with a reduction of VWF ristocetin cofactor and FVIII:C. Generally it is preferable to avoid anticoagulation in patients with VWD due to increased risk of bleeding. However in view of the patient's young age and previous homograft root replacement it was felt unwise to consider further, potentially multiple, redo homograft root replacements, and a mechanical valve was the prosthesis of choice. Our pre-operative evaluation of the patient required a 3-month period of observation whilst on warfarin to ensure that anti-coagulation could be controlled without major problems.

Our case demonstrates that complex cardiac surgery can be performed in patients with underlying congenital coagulopathy, and that a successful outcome requires close multidisciplinary cooperation in terms of planning and monitoring peri-operative factor replacement therapy, the dilemma regarding the type of prosthetic valve and the level of anticoagulation required post-operatively.

## Consent

Written informed consent was obtained from the patient for publication of this case report. A copy of the written consent is available for review by the Editor-in-Chief of this journal.

## Competing interests

The authors declare that they have no competing interests.

## Authors' contributions

KS participated as first assistant in the operation, carried out the study, and wrote the initial manuscript, UB was involved in anaesthetising the patient and collected the relevant literatures, SM anaesthetised the patient and reviewed the manuscript before submission, JA was involved in pre- and post-operative haematology care, revised and corrected the manuscript, ETB performed the operation, revised and corrected the manuscript. All authors read and approved the final manuscript.
